# Ventral Striatal Activation During Reward Anticipation of Different Reward Probabilities in Adolescents and Adults

**DOI:** 10.3389/fnhum.2021.649724

**Published:** 2021-04-20

**Authors:** Maria Bretzke, Hannes Wahl, Michael M. Plichta, Nicole Wolff, Veit Roessner, Nora C. Vetter, Judith Buse

**Affiliations:** ^1^Department of Child and Adolescent Psychiatry, Faculty of Medicine, Technische Universität Dresden, Dresden, Germany; ^2^Institute of Neuroradiology, Faculty of Medicine, Technische Universität Dresden, Dresden, Germany; ^3^Department of Psychiatry, Psychosomatic Medicine and Psychotherapy, University Hospital, Goethe University, Frankfurt, Germany

**Keywords:** adolescence, development, fMRI, reward probabilities, reward, ventral striatum

## Abstract

Adolescence has been linked to an enhanced tolerance of uncertainty and risky behavior and is possibly connected to an increased response toward rewards. However, previous research has produced inconsistent findings. To investigate whether these findings are due to different reward probabilities used in the experimental design, we extended a monetary incentive delay (MID) task by including three different reward probabilities. Using functional magnetic resonance imaging, 25 healthy adolescents and 22 adults were studied during anticipation of rewards in the VS. Differently colored cue stimuli indicated either a monetary or verbal trial and symbolized different reward probabilities, to which the participants were blinded. Results demonstrated faster reaction times for lower reward probabilities (33%) in both age groups. Adolescents were slower through all conditions and had less activation on a neural level. Imaging results showed a three-way interaction between age group x condition x reward probability with differences in percent signal change between adolescents and adults for the high reward probabilities (66%, 88%) while adolescents demonstrated differences for the lowest (33%). Therefore, previous inconsistent findings could be due to different reward probabilities, which makes examining these crucial for a better understanding of adolescent and adult behavior.

## Introduction

Adolescence is a developmental period associated with changes in tolerance of uncertainty (Tymula et al., 2012; van den Bos and Hertwig, 2017). Adolescents are often involved in risky behaviors and higher sensation and novelty seeking (van Duijvenvoorde et al., 2016). This increased attentiveness to sensations and novelty paired with a heightened tolerance of uncertainty may in turn be connected to an increased reward response, leading to greater motivated behavior among adolescents to receive rewards (Braams et al., 2015). At the neural level, altered reward processing has been observed in reward related structures such as the ventral striatum (VS), anterior insula (AI), thalamus and supplementary motor cortex (van Duijvenvoorde et al., 2016; Oldham et al., 2018). Focusing on the VS, research into adolescents' altered sensitivity during reward anticipation has produced inconsistent findings, including both adolescent hyper- (Silverman et al., 2015) and hypoactivation (Bjork et al., 2010). However, this appears to be highly task dependent.

One key paradigm to investigate VS reward processing is the monetary incentive delay (MID) task (Knutson et al., 2000). This paradigm allows one to temporally disentangle the phases of reward anticipation and receipt (Lutz and Widmer, 2014). During these phases similar brain regions, including the VS, pallidum, insula, thalamus, hippocampus, and motor areas (Oldham et al., 2018; Cao et al., 2019) are active in adults and adolescents. However, when contrasting adolescents and adults the activation pattern in the VS varies across studies. While Bjork and colleagues (Bjork et al., 2004, 2010) detected reduced activation of the VS in adolescents compared to adults for cues that predicted a reward, other studies using a different task design observed heightened activation of the VS in adolescents (Ernst et al., 2005; Van Leijenhorst et al., 2010). Overall, the observed hypo- or hyperactivation of the VS in adolescents could signify the reorganizing of the reward and motivation circuity during adolescence (Fair et al., 2009; Doremus-Fitzwater and Spear, 2016). This might reflect adolescent hypersensitivity to larger rewards (Cohen et al., 2010) or different reaction times and neural activations in accordance to different reward probabilities. Previous inconsistent findings of adolescent VS hypo- or hyperactivation may depend on methodical differences between studies such as the task, e.g., MID, wheel of fortune, slot machine task, cake gambling task, pirate task (Jarcho et al., 2012), child-friendly versions of the MID task (Helfinstein et al., 2013; Kappel et al., 2013), or differences in stimuli or context (Nees et al., 2012; Bartra et al., 2013; Nelson et al., 2016).

Few studies have paid attention to different reward probabilities or success rates used within paradigms (Abler et al., 2006; Van Leijenhorst et al., 2010). However, when taking into account that adolescence is associated with changes in the tolerance of uncertainty, risky behaviors and novelty seeking, it can be assumed that differences between adolescents and adults in activation of the VS may also depend on the anticipated probability of a reward. The rate at which a reward is given or a response is successful could therefore play an important role when trying to understand adolescent behavior, in clinical as well as in non-clinical samples. To the best of our knowledge, the relation between activation of the VS as a function of different reward probabilities has never been studied in healthy adolescents compared to healthy adults using the MID task. However, implications for the relevance of taking different reward probabilities into account comes from studies on adolescents with ADHD. While von Rhein and colleagues (von Rhein et al., 2015) chose a 33% reward probability and did not find alterations in reward related regions during reward anticipation in adolescents and young adults with ADHD compared to individuals without ADHD (von Rhein et al., 2015), other studies used a different reward probability of 66% and reported reduced VS activation in adolescents with ADHD compared to those without ADHD (Scheres et al., 2007). A meta-analysis further reported hyporesponsiveness of the VS in adolescents and adults with ADHD compared to those without ADHD (Plichta and Scheres, 2014). These inconsistent findings highlight the importance of different reward probabilities when examining neural reward processing in adolescents.

Thus, the purpose of the current study was to examine and compare the extent to which activation in the VS between adolescents and adults is a function of the respective reward probability. The original MID task (Knutson et al., 2000) uses an adaptive algorithm to achieve an average hit rate of 66%, since a reward probability of 50% induces the highest dopamine release in animal studies (Fiorillo et al., 2003). A reward probability of 66% therefore induces sufficiently high dopamine release and activation in the VS, setting up a positive association between cue and reward (Plichta and Scheres, 2015). This in turn leads to participants' perception of the paradigm as plausible. We extended the original MID paradigm by three different probabilities which varied with respect to the degree a successful reward was gained (i.e., reward probability: 33, 66, and 88%), thus providing a varying degree of uncertainty during reward anticipation.

By including three reward probabilities and comparing adolescents and adults, this opens up the possibility to further investigate the relationship between brain activation in the VS and varying reward probabilities in a developmental context.

We hypothesize, that both age groups will show a higher percent signal change in the VS during the anticipation of the monetary compared to a verbal feedback condition in all reward probabilities. The following hypotheses are referring to the monetary condition. Based on previous work investigating dopamine release in the VS (Fiorillo et al., 2003), we hypothesize that the highest percent signal change and fastest reaction times will occur in the 66% reward probability and a lower percent signal change as well as slower reaction times will occur for the other two reward probabilities in both age groups. However, we expect differences between the age groups for the 33% and the 88% reward probabilities. Specifically, we hypothesize that adolescents demonstrate a higher percent signal change in the VS and faster reaction times compared to adults for the 33% reward probability because adolescents anticipate even unlikely rewards as an incentive. In contrast, compared to adolescents, adults will show faster reaction times and higher percent signal change for the 88% reward probability.

## Materials and Methods

### Participants

This study was part of a larger project dealing with the reward system. For this, we recruited healthy adolescents (10–18 years) and adults (19–45 years) through online advertisement and the databank of the Department of Child and Adolescent Psychiatry of the University Hospital Dresden.

Participants were screened for psychiatric disorders using the MINI/MINI Kid (Sheehan et al., 2010) and had normal or corrected to normal vision. Exclusion criteria included: Left-handedness as measured by The Edinburgh Handedness inventory (Oldfield, 1971), intelligence below-average (IQ < 85) as measured by the Zahlen-Verbindungs-Test (Oswald, 2016; a feasible measure of information-processing speed in which participants connect circled numbers ascending from 1 to 90), color blindness, structural brain abnormalities or a history of psychiatric or neurological disorders and MRI contraindications. 26 adults and 29 adolescents participated. Eight subjects were excluded because of (1) a high error rate (more than 33% errors in any condition, *n* = 1 adolescent, *n* = 1 adult) or (2) technical difficulties (*n* = 1 adolescent) or for matching reasons regarding IQ and gender (*n* = 2 adolescents*, n* = 3 adults). An equivalence test was performed as a cross-check for sex (Cramer's V = 0.015, *p* < 0.920) with a 90% CI and used the TOST procedure for non-verbal IQ [*t*_(45)_ = 1.59; *p* = 0.118]. Error trials were defined as reaction times faster than 100 ms or trials in which the participants did not react to the cue stimulus. A 2x2x3 mixed ANOVA with the factors age group (adolescents vs. adults), condition (monetary vs. verbal) and reward probability (33% vs. 66% vs. 88%) revealed main effects of age group [*F*_(1, 45)_ = 6.7, *p* = 0.013, *partial* η^2^ = 0.129] with more errors for adolescents (M_adolescents_ = 2.13 ± 1.50; M_adults_ = 1.22 ± 0.74), of condition [*F*_(1, 45)_ = 13.15, *p* = 0.001, *partial* η^2^ = 0.226] with more errors in the monetary- compared to the verbal condition and an interaction between condition x reward probability with most errors in the 33% monetary condition [*F*_(2, 90)_ = 3.8, *p* = 0.026, *partial* η^2^ = 0.078; for detailed information, please see Supplementary Table 1]. There were no further effects [effect of reward probability [*F*_(2, 90)_ = 0.539, *p* = 0.585, *partial* η^2^ = 0.012] interaction age group × condition [*F*_(1, 45)_ = 0.286, *p* = 0.596, *partial* = η^2^ = 0.006]; interaction age group × reward probability [*F*_(2, 90)_ = 0.775, *p* = 0.464, *partial* η^2^ = 0.017; three-way interaction age group × condition × reward probability (*F*_(2, 90)_ = 1.3, *p* = 0.278, *partial* η^2^ = 0.028)].

The final sample consisted of 25 adolescents (*M*_*age*_ = 15.45 years ± 2.07; *range*: 10.46–18.90 years) and 22 adults (*M*_*age*_ = 26.96 years ± 3.42; *range*: 22.05–32.03 years; see Table 1). The pubertal developmental Scale (PDS; Petersen et al., 1988); German version (Watzlawik, 2009) was obtained to assess the pubertal status of the adolescents. The PDS scale ranges from 1 (prepubertal-) to 5 (postpubertal status). The adolescents reported to have a mid- to late pubertal status (*M* = 3.64 ± 0.86, *range:* 2–5; see Table 1 for details).

**Table 1:** Participant characteristics (*n* = 47).

	**Adolescents (*****n*** **=** **25)**		**Adults (*****n*** **=** **22)**		**Comparison between age groups**			**90% CI**
	**M (SD)**	**range**	**M (SD)**	**range**	***t***	***df***	***p***	
Age in years	15.40 (2.10)	10.50–18.90	26.96 (3.42)	22.05–32.03	14.14^a^	33.7	<0.001	[10.147–12.88]
Number of females	14		12					
Non-verbal IQ^*^	112.10 (13.94)	94.00–143.50	117.80 (10.20)	101.50–145.00	1.59	45	0.118	[−0.317–11.79]
Money received in €	25.92 (1.90)	22.00–29.50	27.32 (1.68)	24.00–30.00	2.65	45	0.011	[0.515–2.28]

* *To estimate the processing speed component of intelligence we used the Zahlen-Verbindungs-Test (Oswald, 2016)*.

a *Levene's test is significant (p < 0.05), suggesting a violation of the assumption of equal variances*.

Participants were reimbursed monetarily for their participation and gained as a mean payoff 26.57 € (*SD* = 1.91; *range:* 22–30). Written informed consent was obtained from participants and their legal guardians before testing. The study was carried out following the latest guidelines of the Declaration of Helsinki and approved by the local ethics committee of the Medical Faculty of the TU Dresden.

### Paradigm, Design, and Procedure

**Figure 1:**
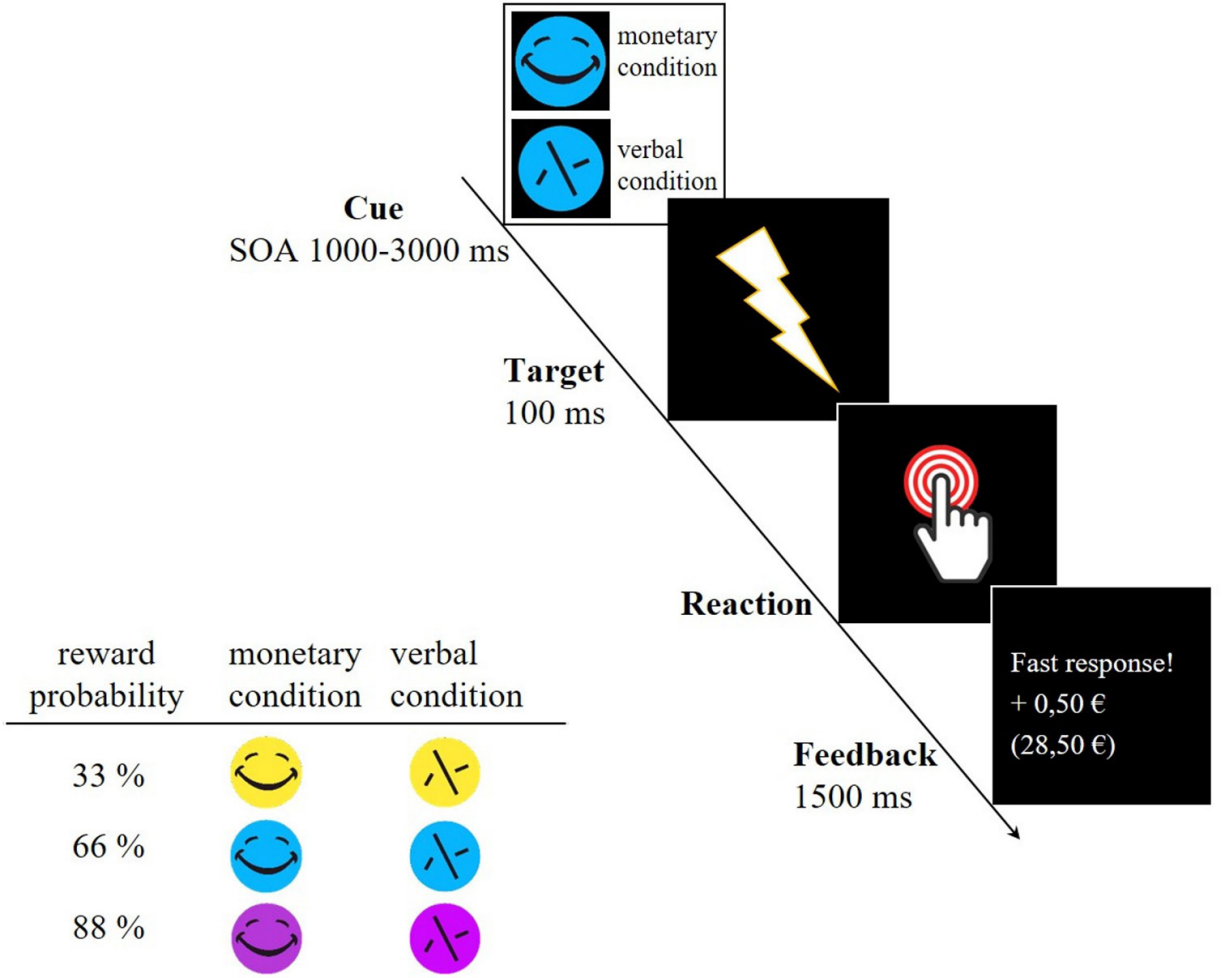
Example trial of the MID task and reward probabilities indicated by differently colored cue stimuli. A smiley represented monetary trials whereas a scrambled face symbolized the control trial in which participants only received verbal feedback. SOA, Stimulus Onset Asynchrony.

The participants underwent a modified version of the original monetary incentive delay (MID) task that reliably elicited VS activity (Plichta et al., 2012). This version was supplemented by three different reward probabilities (see Figure 1). First, a cue stimulus (CS) indicated either a monetary trial (smiley, possible win of 0.50 €) or a verbal trial which acts as a control trial (scrambled smiley, verbal feedback). The CS appeared in three different colors, indicating different reward probabilities (yellow = 33%, blue = 66% and pink = 88%). Shortly after the CS (SOA 1,000–3,000 ms, mean 2,000 ms) the participants saw a flash (target) to which they had to respond as fast as possible by pressing a button with the right index finger. After each trial, the valid reaction time window (RT window) for the next trial was adapted to the individual reaction time in order to set the different reward probabilities. In the beginning, the maximum reaction time for each reward probability was set as follows: 33%−200 ms, 66%−300 ms, and 88%−400 ms. The example of the 33% reward probability illustrates how the intended probabilities were maintained. In this case, participants had to react within a RT window with a maximum of 200 ms in the beginning. If participants reacted within the RT window the trial was successful and unsuccessful if they did not reach the reaction time window or did not react. In the further course, this RT window was adapted as followed:

$$\begin{array}{l} \text{Successful }:\text{ RTwindow }=\text{ RTwindow}-\;\left(\text{RTwindow }*\;0.07\right)\\ \text{Unsuccessful }:\text{ RTwindow }=\text{ RTwindow}+\;\left(\text{RTwindow }*\;0.03\right) \end{array}$$

This ensured a reward probability of 33%. For the 66% reward probability, the RT window was multiplied by 0.05 in both cases and for the 88% reward probability by 0.03 (successful) and 0.07 (unsuccessful). This adaptation of the RT window also guaranteed that each individual gained nearly the same amount of money by the end of the experiment (average 27 €). Participants received feedback after each trial, disclosing whether the response was fast enough and how much money had been gained. Each cue stimulus appeared 30 times, summing up to 180 trials in total. The scanning procedure of the paradigm took about 22 min for each participant. After performing the task, the participants ranked the different reward probabilities represented by differently colored stimuli with regard to their success rate. After evaluating the ranking, no differences occurred between adolescents and adults [33%: M_adolescents_ = 2.04 ± 0.91; M_adults_ = 1.86 ± 0.96; *t*33%_(43)_ = −0.661, *p* = 0.512; 66%: M_adolescents_ = 2.13 ± 0.68; M_adults_ = 1.95 ± 0.74; *t*66%_(43)_ = −0.816, *p* = 0.419; 88%: M_adolescents_ = 1.83 ± 0.87; M_adults_ = 2.19 ± 0.75; *t*88%_(43)_ = 1.47, *p* = 0.150] as well as between the different reward probabilities within each age group [Adolescents: *t*33_66_(23)_ = −0.303, *p* = 0.765; *t*33_88_(23)_ = 0.622, *p* = 0.540; *t*66_88_(23)_ = 1.13, *p* = 0.271; Adults: *t*33_66_(20)_ = −0.282, *p* = 0.781; *t*33_88_(20)_ = −0.979, *p* = 0.339; *t*66_88_(20)_ = −0.960, *p* = 0.348]. One adolescent and one adult could not rank the different stimuli.

Task presentation and recording of the behavioral responses were performed using Presentation® software (Version 20.1 Build 12.04.17, Neurobehavioral Systems, Inc., Albany, CA). Reaction times were collected with ResponseGrips (©NordicNeuroLab). The scanning session was instantly preceded by a practice session outside the scanner and participants got to practice 20 trials. The stimuli used in that session differed in their order of occurrence from those in the actual task. Participants were shown a box of cash before the scanning session.

### Analysis of Behavioral Data

To analyze the behavioral data, SPSS (Version 26) for Windows was utilized for mean reaction times (RTs). Each trial in which participants did not response or reacted faster than 100 ms was considered as an error trial and discarded from the analysis. By further computing mean values and standard deviations for each participant, we only included values within a mean ± 2 SD range for each individual in the analysis of the reaction times. A 2 × 2 × 3 ANOVA was calculated with a threshold of *p* < 0.05 with the between-subject factor *age group* (adolescents vs. adults) and the two within-subject factors *condition* (monetary vs. verbal) and *reward probability* (33 vs. 66 vs. 88%). Whenever necessary, *post-hoc* tests were Scheffé-corrected.

### Functional Imaging

#### Image Acquisition

MRI scans were acquired using a 3-tesla whole body scanner (MAGNETOM Prisma, Siemens Medical Solutions) equipped with a 64 channel brain array coil. We used T2^*^-weighted single shot gradient echo planar imaging (EPI) sequences that were motion corrected using prospective acquisition correction (PACE) technique to acquire fMRI data (TR/TE = 3,000/30 ms, FOV = 192 × 192 mm, 49 axial slices, 2 × 2 × 2 mm voxel size, flip angle = 90°). The first five EPI volumes were rejected to allow for T1 equilibration. Following the functional acquisition, a high resolution 3D T1-weighted magnetization-prepared rapid gradient echo (MPRAGE) was conducted (TR/TE = 2,400/2.23 ms, FOV= 272 × 272 mm, 240 sagittal slices, 0.85 × 0.85 × 0.85 mm isotropic voxel size, flip angle = 8°). Those specified parameters provided a whole brain coverage with only omitting the inferior part of the cerebellum. The paradigm was presented on a MR-suitable LC-Display behind the scanner bore. Participants were able to see the stimuli through a mirror attached to the head coil. Functional images were acquired before the anatomical images. All anatomical data were screened for clinical abnormalities by a neuroradiologist. Structural and functional data were scanned for motion artifacts in accordance to Backhausen et al. (2016).

#### Analysis of fMRI Data

##### Preprocessing

Functional imaging data were analyzed and preprocessed using the statistical parametric mapping toolbox 12 (SPM12v7487; Wellcome Trust Centre for Neuroimaging, London, UK). The preprocessing pipeline included (1) slice-time correction using the middle slice of the volume as reference, (2) rigid realignment with 6 degrees of freedom to the first volume and unwarp for motion correction. Maximum participant movement at each time point in any direction did not exceed 2.5 mm or 2.5 degrees and no participant was excluded due to excessive movement, (3) A rigid coregistration with 6 degrees of freedom of the individual anatomical image to the mean functional image was performed and followed by (4) segmentation of the coregistered (not resliced) anatomical image, to calculate the necessary spatial normalization and the brain mask for the first-level analysis. The resulting non-linear spatial normalization into Montreal Neurological Institute [MNI] space was (5) applied to the functional images which were finally (6) spatially smoothed with a Gaussian kernel with a full-width half-maximum (FHWM) of 8 mm.

##### Statistical Analyses

The first-level analysis was done within a GLM framework by modeling six regressors of interest: One factor *condition* with two levels, (1) monetary and (2) verbal condition, and another factor *reward probability* including three levels (3) 33%, (4) 66% and (5) 88%. These were modeled at the point of presentation as stick functions convolved with a standard canonical haemodynamic response function (HRF). Notably, trials with missings or wrong responses (e.g., pressing the button before the flash or reaction <100 ms) and trials that included reaction times diverging from mean ± 2SD were included in the model as a regressor of no interest (M_missings_ = 1.71 ± 1.28; see Supplementary Table 1 for details). Mean values and standard deviations were calculated for each participant across all trials. In addition, the onsets of the target stimulus *flash* and the response, the onsets of the feedback as well as the six subject-specific movement regressors from the rigid-body realignment were included as regressors of no interest. Analysis was restricted to the gray and white matter, excluding the ventricles by using the individual normalized brain masks to account for differences in brain sizes between our age groups. A high-pass filter set at 128 s was used to attenuate low-frequency components of physiological noise (Henson, 2004).

To investigate age-related activation differences in the reward anticipation phase that could be modulated by different reward probabilities, we performed a whole-brain analysis with a 2 × 2 × 3 full factorial model including the same factors used for the analysis of the behavioral data. Brain coordinates are reported in MNI atlas space. Contrasts were thresholded at *p* < 0.05 FWE-corrected level. If this rather conservative correction showed no effects, clusters would be reported on the cluster corrected threshold criterion of *p* < 0.05 using the preselection threshold of *p* < 0.001 on a voxel level and an extent threshold ensuring a minimum cluster size of 10 voxels.

##### Region of Interest Analysis of the VS

Based on the literature and the results of our whole brain analysis, we utilized a Region-of-interest (ROI) approach focusing on the left and right side of the VS. Previous findings suggested a biased effect of the smoothing kernel size on the spatial localization of striatal activity foci (Sacchet and Knutson, 2013). Therefore, we decided to use the coordinates for a smoothing kernel >7 mm resulting from an Activation Likelihood Estimate (ALE) meta-analysis (Sacchet and Knutson, 2013). We transformed the given Talairach - into MNI coordinates by using the Yale BioImage Suite tool tal2mni (Lacadie et al., 2008) and surrounded the resulting coordinates with a 7 mm Sphere for use as ROIs of the left and right VS.

We extracted percent signal change of left and right VS with rfxplot (Gläscher, 2009) and computed a 2 × 2 × 3 mixed ANOVA with the factors described above. Since the age gap between the two age groups is relatively small, we also took a dimensional approach in analyzing the predictive effect of age on the percent signal change in the VS. We conducted quadratic regression analyses with age as predictor variable and the difference in percent signal change between monetary and verbal condition in bilateral VS as dependent variable. One quadratic regression was conducted for the total value and three additional regressions for the respective reward probabilities to examine the predictive effect of age on varying reward probabilities. The difference value was calculated by subtracting the percent signal change in the verbal condition from that in the monetary condition for the left and right VS separately. Then we calculated the mean between the difference scores of the left and right VS, resulting in one score for the bilateral VS. Therefore, when using the term bilateral VS in the following text, we refer to the mean value of left and right VS. *Post-hoc* tests were Scheffé-corrected if necessary.

## Results

### Behavioral Results

#### Reaction Times

**Figure 2:**
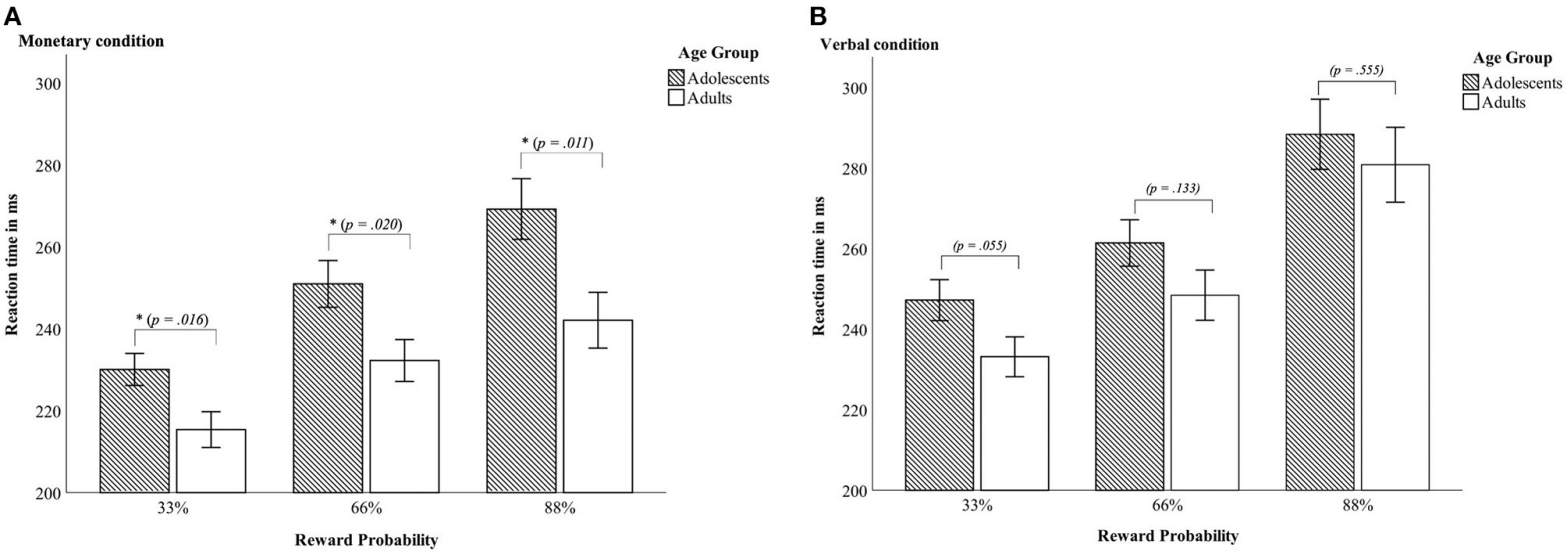
Reaction times—Interaction between age group x reward probability in the monetary condition **(A)** and in the verbal condition **(B)**. Error bars denote ± 1 SE. *p ≤ 0.05.

A 2 × 2 × 3 mixed ANOVA with reaction time as dependent variable revealed a main effect of condition [*F*_(1, 45)_ = 108.71, *p* < 0.001, *partial* η^2^ = 0.707], with both age groups being faster in the monetary condition compared to the verbal condition. Further, there was a main effect of reward probability [*F*_(2, 90)_ = 115.62, *p* < 0.001; *partial* η^2^ = 0.720] with greatest differences between the 33% and 88% reward probability [*t*33_66 _(46)_ = 11.46, *p* < 0.001, *d* = −1.67; *t*33_88 _(46)_ = −12.06, *p* < 0.001, *d* = −1.76; *t*66_88 _(46)_ = 8.54, *p* < 0.001, *d* = 1.25; see Table 2 and Figure 2 for details]. Data indicated a trend toward a main effect of age group [*F*_(1, 45)_ = 3.86, *p* = 0.056 *partial* η*2* = 0.079] pointing in the direction of generally slower reaction times in adolescents (M_adolescents_ = 257.86 ± 29.02; M_adults_ = 242.00 ± 25.91). There was a two-way-interaction age group × condition [*F*_(2, 45)_ = 5.11, *p* = 0.029 *partial* η^2^ = 0.102] with adults being faster than adolescents in the monetary but not in the verbal condition (see Table 2 and Figure 2). Additionally, there was an interaction of condition × reward probability [*F*_(2, 90)_ = 14.7, *p* < 0.001 *partial* η^2^ = 0.246] demonstrating that the difference between the varying reward probabilities was larger in the verbal condition.

**Table 2:** Behavioral results: reaction times for both age groups (*n* = 47).

	**Adolescents *n* = 25**	**Adults *n* = 22**	***Post-hoc*** **tests–comparison between groups**				
	**M (SD) in ms**	**M (SD) in ms**	**Mean difference**	***t***	***df***	***p_***Scheffe***_***	***d***
**Total**	257.86 (29.02)	242.00 (25.91)	−15.85	−1.96	45	0.056	−0.574
Monetary condition	250.10 (27.10)	230.02 (23.99)	−20.06	−2.67	45	0.010	−0.781
Verbal condition	265.62 (31.97)	254.25 (29.55)	−11.37	−1.26	45	0.214	−0.368
	*t*_(24)_ = 6.6; *p* < 0.001	*t*_(21)_ = 7.9; *p* < 0.001					
**Reward probability 33%**							
Total	238.61 (22.10)	224.25 (21.41)	−14.36	−2.26	45	0.029	−0.660
Monetary condition	230.02 (19.54)	215.37 (20.48)	−14.66	−2.51	45	0.016	−0.733
Verbal condition	247.20 (25.40)	233.13 (23.27)	−14.06	−1.97	45	0.055	−0.576
	*t*_(24)_ = −8.4; *p* < 0.001	*t*_(21)_ = −8.9; *p* < 0.001					
**Reward probability 66%**							
Total	256.16 (27.87)	240.32 (25.80)	−15.85	−2.01	45	0.050	−0.588
Monetary condition	250.93 (28.56)	232.22 (24.07)	−18.72	−2.41	45	0.020	−0.705
Verbal condition	261.39 (28.85)	248.41 (29.24)	−12.98	−1.53	45	0.133	−0.447
	*t*_(24)_ = 3.8; *p* = 0.001	*t*_(21)_ = 5.3; *p* < 0.001					
**Reward probability 88%**							
Total	278.79 (39.37)	261.43 (34.84)	−17.36	−1.59	45	0.119	−0.465
Monetary condition	269.21 (37.19)	242.06 (31.91)	−27.14	−2.67	45	0.011	−0.780
Verbal condition	288.38 (43.48)	280.81 (43.53)	−7.568	−0.60	45	0.555	−0.174
	*t*_(24)_ = −5.2; *p* < 0.001	*t*_(21)_ = −5.8; *p* < 0.001					

Last, there was a three-way-interaction age group × condition × reward probability [*F*_(2, 90)_ = 5.4, *p* = 0.006 *partial* η^2^ = 0.107]. Thus, differences between adolescents and adults occurred only in the monetary condition and were most pronounced in the 88% reward probability (see Table 2 and Figure 2).

### fMRI Results

#### Whole Brain Analysis

##### Effects of Condition

The following values were FWE-corrected at *p* < 0.05 on a whole brain level. As previously demonstrated (Wilson et al., 2018; Cao et al., 2019), higher activation was found in the following regions during the anticipation of the monetary compared to the verbal condition: Right and left VS (left: x, y, z = −12, 8, −4; *k* = 56271, *z* > 8; right: x, y, z = 12, 10, −4, *z* > 8, part of the same cluster), right supplementary motor cortex (x, y, z = 4, 8, 52, *z* > 8, part of the same cluster), right middle frontal gyrus (x, y, z = 34, 48, 26; *k* = 823, *z* = 7.37) and right middle temporal gyrus (x, y, z = 50, −26, −8; *k* = 49, *z* = 5.44). The contrast verbal > monetary condition elicited activation in the left angular gyrus (left: x, y, z = −50, −66, 34; *k* = 287, *z* = 6.77), left superior frontal gyrus (x, y, z = −14, 42, 50; *k* = 120, *z* = 5.79) extending to the left middle frontal gyrus (x, y, z = −30, 22, 52, *z* = 5.04) and the left precuneus (x, y, z = −4, −56, 34; *k* = 84, *z* = 5.68).

##### Effects of Reward Probability

After family wise error (FWE) correction, no main effect of reward probability occurred. The following values are therefore reported on a threshold of *p* < 0.05 correction on the cluster level. Results showed activation in the left and right caudate, right cerebellum exterior, occipital gyri (left superior, left inferior, left middle and right inferior; for details, see Table 3). The same threshold was used for the interaction condition × reward probability and this interaction elicited activation in the right opercular part of the inferior frontal gyrus.

**Table 3:** Functional activity associated with the main effect of probability (*n* = 47).

**Brain region**	**L/R**	**Peak-voxel (mm)**			***F*-value**	**Cluster-corrected *p*-value**	**Cluster size *k***
		***x***	***y***	***z***			
Caudate	L	−14	20	−6	14.63	0.003	131
	R	12	20	−2	12.12	0.043	52
Superior occipital gyrus	R	28	−70	16	13.04	0.001	164
	L	−10	−88	26	11.76	0.001	193
Calcarine cortex	R	22	−76	6	8.60		Part of same cluster
Cerebellum exterior	R	48	−64	−30	12.53	0.001	180
Occipital pole	L	−4	−96	22	8.96		Part of same cluster
Cuneus	R	4	−86	24	8.37		Part of same cluster
Inferior occipital gyrus	L	−50	−72	−8	11.33	0.037	56
	R	50	−72	0	9.39	0.043	52
Middle occipital gyrus	L	−46	−76	10	10.14	0.004	124

*p < 0.05, corrected cluster level; k > 10*.

##### Developmental Effects

No main effect of age group occurred after FWE correction. As a result, the following values are reported on a threshold *p* < 0.05 correction on the cluster level. Regarding the main effect of age group, no significant activation emerged. The interaction between age group x condition elicited activation in the right anterior insula (x, y, z = 36, 22, 2; *k* = 71, *z* = 3.81), the left superior parietal lobe (x, y, z = −12, −74, 42; *k* = 78, *z* = 3.77) and the left middle occipital gyrus (x, y, z = −44, −70, 12; *k* = 62, *z* = 3.51). There was no activation for the interaction between age group x reward probability and no three-way interaction.

#### Region of Interest Analysis–Ventral Striatum

**Figure 3:**
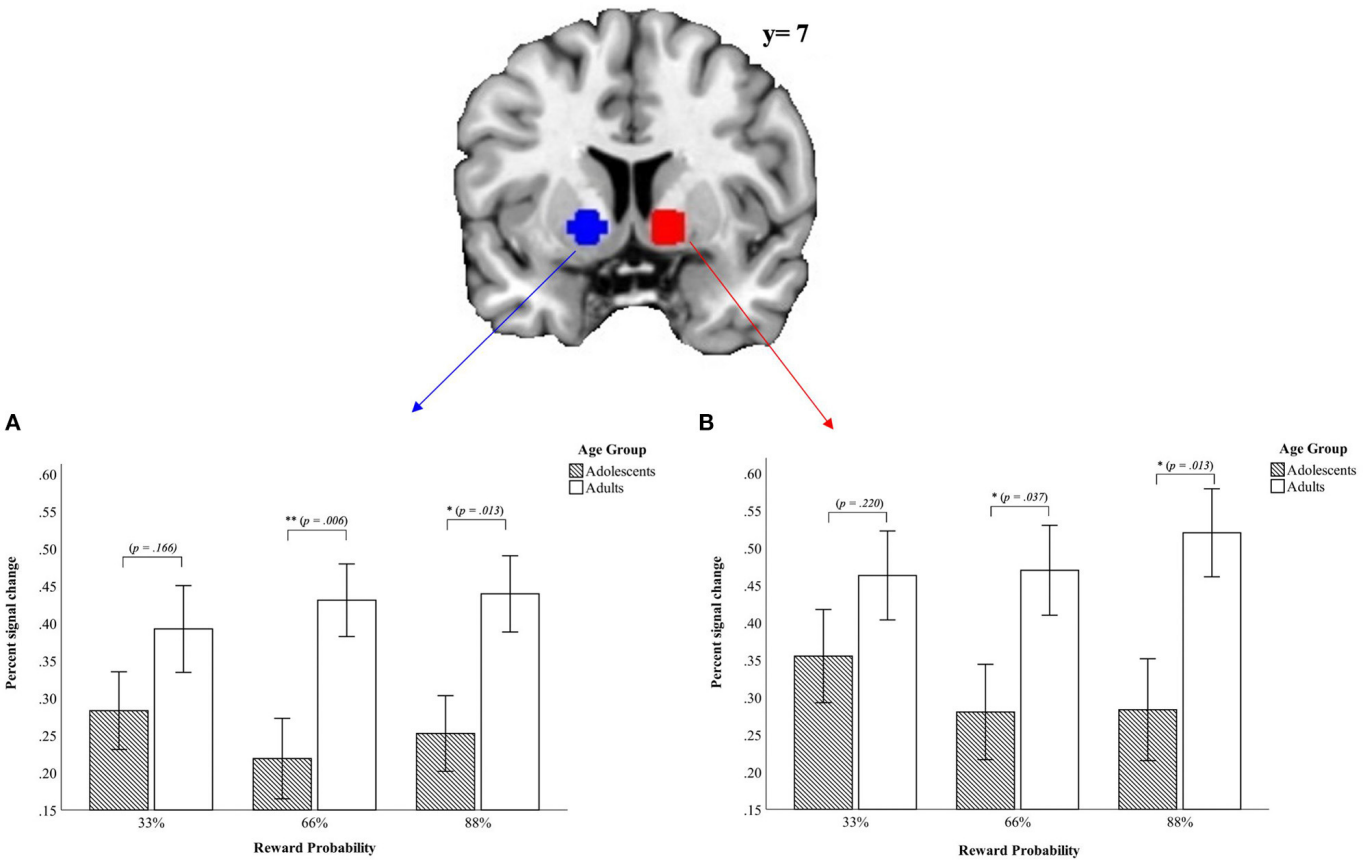
Mean values of percent signal change in the ROI for left **(A)** und right **(B)** VS from the signal difference monetary vs. verbal condition (*p* < 0.05, corrected cluster level, *k* > 10). Error bars denote ± 1 SE. *p ≤ 0.05; **p ≤ 0.01.

First, in the left VS data revealed a *main effect of condition* [*F*_(1, 45)_ = 89.81, *p* < 0.001, *partial* η*2* = 0.666] and a main effect of reward probability [*F*_(2, 90)_ = 8.09, *p* = 0.001, *partial* η^2^ = 0.152] but no main effect of age group [*F*_(1, 45)_ = 0.011, *p* = 0.916, *partial* η^2^ = 0.000]. The *main effect of condition* demonstrated higher activity in the VS during the monetary condition whereas the main effect of reward probability shows that the VS activation varies as a function of the different reward probabilities with the greatest difference in percent signal change in the VS between the 66% and 88% reward probability [*t*33_66%_(46)_ = −0.398, *p* = 0.692; *t*33_88%_(46)_ = −2.99, *p* = 0.004; *t*66_88%_(46)_ = 3.86, *p* < 0.001]. The greatest percent signal change for the left VS occurred in the 66% reward probability (*M*_33%_ = 0.039 ± 0.17; *M*_66%_ = 0.044 ± 0.16; *M*_88%_ = 0.0001 ± 0.20). Further, we found an *interaction* between *age group x condition* [*F*_(1, 45)_ = 5.71, *p* = 0.021, *partial* η^2^ = 0.113] indicating a higher positive signal change in the monetary condition for adults (Monetary condition: *M*_*adults*_ = 0.24 ± 0.19; *M*_*adolescents*_ = 0.15 ± 0.19; Verbal condition: *M*_*adults*_ = −0.18 ± 0.21; *M*_*adolescents*_ = 0.10 ± 0.24). The significant *three-way interaction* age group x condition x reward probability [*F*_(2, 90)_ = 3.69, *p* = 0.029, *partial* η^2^ = 0.076] demonstrated that the signal difference between the verbal condition and the monetary condition comparing adolescents and adults emerged in the 66% and the 88% reward probabilities [*t*33%_(45)_ = 1.41, *p* = 0.166; *t*66%_(45)_ = 2.89, *p* = 0.006; *t*88%_(45)_ = 2.59, *p* = 0.013; see Figure 3]. We did not find other interactions (*p* > 0.558).

Second, for the right VS there was a *main effect of condition* [*F*_(1, 45)_ = 88.43, *p* < 0.001, *partial* η^2^ = 0.663], indicating that the right VS was activated more strongly in the monetary condition as compared to the verbal condition. There was further a main effect of reward probability [*F*_(2, 90)_ = 8.1, *p* = 0.001, *partial* η^2^ = 0.152], driven by the difference between the 66% and 88% and the difference between 33 and 88% reward probability [*t*33_66%_(46)_ = 0.464, *p* = 0.645; *t*33_88%_(46)_ = −3.14, *p* = 0.003; *t*66_88%_(46)_ = 3.26, *p* = 0.002]. The highest percent signal change occurred in the 88% reward probability (*M*_33%_ = −0.016 ± 0.18; *M*_66%_ = −0.02 ± 0.17; *M*_88%_ = −0.06 ± 0.20).

Further, there was a *two-way interaction* between age group x condition [*F*_(1, 45)_ = 4.49, *p* = 0.040, *partial* η^2^ = 0.091]. The effect of the *two-way interaction* age group x condition was driven by a tendency toward greater differences between the two age groups in the verbal condition (Verbal Condition: *M*_*adolescents*_ = −0.18 ± 0.28; *M*_*adults*_ = −0.28 ± 0.21; *t*_verbal(45)_ = −1.36, *p* = 0.182; Monetary condition: *M*_*adolescents*_ = 0.13 ± 0.20; *M*_*adults*_ = 0.20 ± 0.23; *t*_monetary(45)_ = 1.28, *p* = 0.209]. Further, the difference between the monetary and the verbal condition were less pronounced in adolescents than in adults also contributing to the observed effect. Last, there was a *three-way interaction* between age group x condition x reward probability [*F*_(2, 90)_ = 3.44, *p* = 0.039, *partial* η^2^ = 0.071]. This indicated that the differences between the verbal and monetary condition were most pronounced for adults in the 88% reward probability and for adolescents in the 33% reward probability. Adolescents and adults differed for the higher reward probabilities but not for the lowest reward probability of 33% (for details see Figure 3).

**Figure 4:**
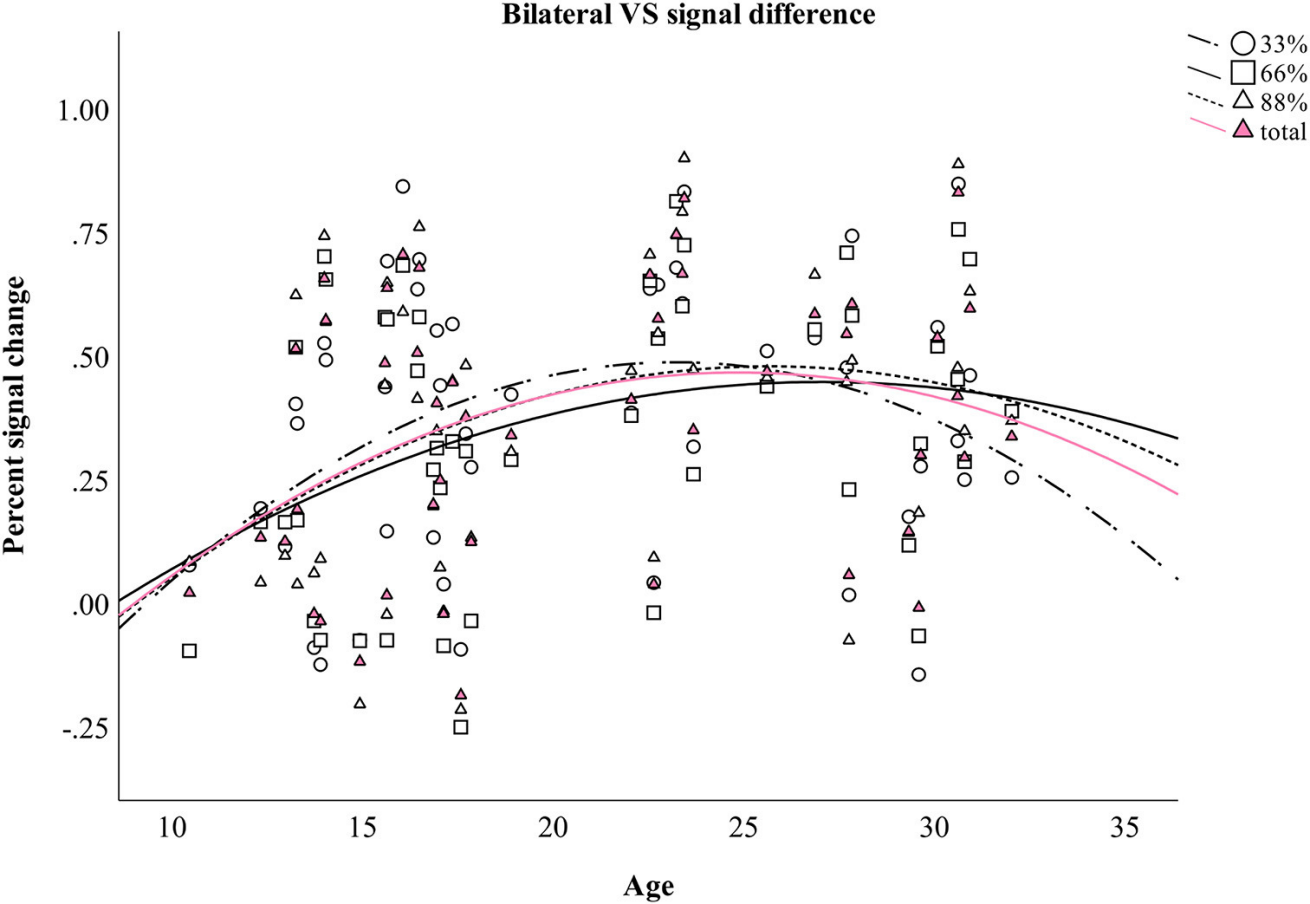
Difference between monetary and verbal condition in the percent signal change in bilateral VS during aging. Individual values as well as regression curves against age are depicted for bilateral VS.

Four additional non-linear regression analyses were conducted to further analyze the relationship between age and the difference in percent signal change in bilateral VS between verbal and monetary condition. Increasing age was associated with an increase in the difference between verbal and monetary condition until the age of ~25 years and declined afterwards (see Table 4 and Figure 4). Analyses conducted for right and left VS separately revealed comparable results.

**Table 4:** Non-linear regressions of the predictor variable (age) on the dependent variable (difference between the monetary and verbal condition) in bilateral VS.

		***B***	***SE B***	**β**	***t***	***p***	**Adjusted *R^**2**^***
**Bilateral VS**							
total	Intercept	−0.684	0.547		−1.250	0.218	
	Age	0.092	0.054	2.188	1.723	0.092	
	Age^2^	−0.002	0.001	−1.933	−1.522	0.135	0.078
33%	Intercept	−0.876	0.555		−1.579	0.122	
	Age	0.117	0.054	2.750	2.158	0.036	
	Age^2^	−0.003	0.001	−2.604	−2.043	0.047	0.070
66%	Intercept	−0.507	0.575		−0.881	0.383	
	Age	0.071	0.056	1.59	1.251	0.218	
	Age^2^	−0.001	0.001	−1.293	−1.016	0.315	0.074
88%	Intercept	−0.668	0.583		−1.147	0.258	
	Age	0.089	0.057	1.978	1.563	0.125	
	Age^2^	−0.002	0.001	−1.693	−1.338	0.188	0.083

*Four separate quadratic regressions were conducted (one for the total value and one for each reward probability separately)*.

## Discussion

This study aimed for a better understanding of reward processing from a developmental perspective by comparing adolescents and adults in terms of their processing of different reward probabilities. Previous studies mostly used a reward probability of 66% as this had been demonstrated to induce the highest dopamine release in reward related structures (Fiorillo et al., 2003; Plichta and Scheres, 2015). Expanding the task by two further reward probabilities, we were particularly interested if and to what extent these different reward probabilities lead to different effects in adolescents as compared adults on the behavioral and neural level. Adolescence is a period of increased tolerance toward uncertainties (Blankenstein et al., 2016) and rather unlikely rewards might be a stronger incentive for adolescents as compared to adults. We therefore expected a difference between both age groups such that adolescents as compared to adults react faster and with a higher percent signal change in the VS for the lowest reward probability (33%) whereas adults demonstrate faster reaction times and higher percent signal change than adolescents for the high reward probabilities (66 and 88%).

We presented participants with a MID task that compared a monetary with a verbal condition and included three different reward probabilities indicated by differently colored cue stimuli. Participants were not informed beforehand that the different colors corresponded to different reward probabilities. After the scanning session, participants were asked to rank the cue stimuli with respect to their anticipated gain. Both age groups did not differ from each other regarding the ranking.

In line with previous work (Plichta et al., 2013), our task induced faster reaction times in the monetary condition for both groups and we found an interaction between age group x condition such that adults were faster than adolescents in the monetary but not the verbal condition. While the whole brain analysis revealed overlapping brain regions including the VS for adolescents and adults in the contrast monetary > verbal condition in line with previous literature (Plichta et al., 2013; Cao et al., 2019), we did not find an interaction effect between age group and condition for VS activation. For this interaction, our data revealed activation in the right anterior insula, left superior parietal lobule and left middle occipital gyrus but not in the VS on a whole brain level. As such, our data did not support results indicating striatal hyperresponsiveness in the VS (e.g., Van Leijenhorst et al., 2010) in adolescents as compared to adults that have been a point of discussion within the research community. Current data illustrate an effect of reward probability on a behavioral and neural level. Both groups reacted faster in response to the low reward probability of 33%, however, on a neural level, differences between age groups emerged. Adolescents demonstrated the greatest percent signal change in the VS for the 33% reward probability, while adults had the greatest percent signal change for the higher reward probabilities (66 and 88%). Previous inconsistent results may have therefore been induced by the different reward probabilities that were used in the task. However, current findings could also be due to the different framing used in our study by not integrating loss trials into the task.

Behavioral data demonstrated a main effect of reward probability. Inherent in the task using three different probabilities were varying speed requirements such that the 88% reward probability started with a reaction time window that was easier to meet than 66%, which in turn was easier than 33%. Therefore, the declining of a higher hit rate and reward probability from 88 to 33% was associated with higher speed requirements which are linked to cognitive effort that has been demonstrated to influence the ventral and dorsal striatum (Schouppe et al., 2014; Dobryakova et al., 2017). Furthermore, the paradigm adapted to the individual reaction times of which the participants were not aware. Therefore, this effect could possibly emerge because different reward probabilities inherent in the MID task imply a different task difficulty through different speed demands to receive a reward. Even in the absence of a reward, previous work demonstrated a critical role of the VS to mobilize cognitive resources necessary to accomplish tasks in a parametric way (Boehler et al., 2011). However, data also revealed a three-way interaction between age group × condition × reward probability. The largest differences between adolescents and adults occurred in the 88% reward probability such that adolescents were indeed slower than adults. However, this only applied to the monetary condition, which could indicate that the current data does not only reflect the mobilization of cognitive resources.

Similar effects were detectable at the neural level. The whole brain analysis revealed for the processing of different reward probabilities a main effect in the bilateral VS. Thus, VS activation differed as a function of different reward probabilities. This corresponds to the findings of Yacubian et al. (2007) who, based on findings in non-human primates (Fiorillo et al., 2003; Tobler et al., 2005), exploited a guessing task to examine the processing of reward probability and reward magnitude in VS subregions and demonstrated greater anterior and lateral peak activation foci in the VS for high and low reward probabilities (Yacubian et al., 2007). However, unlike previous work which dealt with different reward probabilities in the wheel of fortune decision-making task (Smith et al., 2009), we did not inform participants which reward probability was related to which color. Presumably for this reason, we did not find activity in the dorsal anterior cingulate cortex, a structure involved in conflict monitoring (Smith et al., 2009). As the processing of rewards has been linked to the VS, we used this region to conduct a follow-up ROI analysis. In addition to main effects of condition and probability, this analysis also demonstrated a three-way interaction between age group × condition × reward probability for both sides of the VS such that adolescents exhibited the strongest activation for the lowest reward probability (33%) when it was challenging to gain a reward whereas adults demonstrated this for the highest reward probability (88%) when the reward was certain. This points in the direction that adolescents and adults demonstrate a different tolerance toward uncertainty (Luigjes et al., 2016). This could further be linked to regulative aspects of reward-responses and goal-directed behavior. Prefrontal regions like the medial prefrontal cortex (mPFC) are connected with the VS and form striatal cortical loops (Haber and Knutson, 2010) that support reward related learning (Cox and Witten, 2019). Given the maturational imbalance model (Casey et al., 2008) one would expect altered prefrontal activation when comparing adolescents and adults. And indeed, previous studies demonstrated that during development the interconnection between mPFC and VS is stronger at younger ages and decreases with age (Fareri et al., 2015). However, not in line with previous results, our data did not reveal differences in prefrontal regions on a whole brain level when looking at developmental differences. That being said, examining the interplay between prefrontal regions and the VS is important for understanding adolescent reward processing and should be incorporated in future studies.

In addition, adolescents demonstrated smaller differences between the verbal and the monetary condition. Previous research has compared the processing of monetary and social rewards in children, adolescents and adults and came to the conclusion that while both social and monetary rewards induced faster reaction times in all age groups, social rewards demonstrated a higher incentive than monetary rewards in adolescents (Ethridge et al., 2017; Wang et al., 2017). Therefore, current findings could be influenced in such a way that the verbal condition acts as a social reinforcer and has greater impact on adolescents than on adults. However, such findings seem further to be highly dependent on the reward probability used in the paradigm. The differences between the verbal and monetary condition were greatest in the 33% reward probability in adolescents and greatest for the 88% reward probability in adults. This emphasizes not only the two conditions being anticipated differently but also that this difference depends on the probability to gain a reward influencing adolescents and adults in a different manner. This could illustrate a developmental shift from adolescence to adulthood. Greimel et al. further highlighted the differences between male and female adolescents (Greimel et al., 2018) which also needs to be considered in future studies. Previous research demonstrated an influence of hormone levels (Op de Macks et al., 2011) and pubertal status on brain activation (Forbes and Dahl, 2010; Urošević et al., 2014). Adolescents in other studies that compared adolescents and adults (e.g., Bjork et al., 2004) might have had different pubertal status than the current sample. Therefore, current results might not be generalized for all adolescents as they only reflect mid- to late pubertal status. Studies that include the puberty status as a possible covariate and assess reward processing dimensionally through adolescence are needed.

Current data are inconsistent with the hypothesis of a reversed U-shape activity in the VS previously demonstrated in non-human primates (Fiorillo et al., 2003). We did not find this activation pattern for adolescents and adults. Instead we observed an increase of VS activation with reward increasing probability in adults, and a decrease in VS activation from lower to higher reward probabilities in adolescents. The observed increase of activation in the VS in adults is more in line with previous work (Abler et al., 2006) in which the participants saw the respective probabilities indicated on a pie chart. In addition, other studies also found a linear increase in VS activity for reward magnitude (0.20 $ vs. 1 $ vs. 5 $) with a greater increase in adults than in adolescents (Vaidya et al., 2013).

Reward sensitivity is not only of interest for a better understanding of adolescent behavior (Ernst et al., 2005; Bjork et al., 2010) but also allows for a better understanding of the development of mental health problems. Previously, reward related VS activation has been discussed in the context of different psychiatric disorders, e.g., Tourette syndrome, Eating Disorders, addiction disorders or ADHD (Paloyelis et al., 2012; Balodis and Potenza, 2015; Matton et al., 2017; Akkermans et al., 2019). However, varying reward probabilities used in the paradigms may have led to inconsistent findings in psychiatric populations, e.g., ADHD which ranges from no differences in VS activation (von Rhein et al., 2015) to hypoactivation in the VS for patients with ADHD (Plichta and Scheres, 2014). Examining the association between psychiatric disorders and reward sensitivity as a function of different degrees of uncertainty could broaden our understanding of disorders that have been associated with harm avoidance (Hauser et al., 2016) as well as a reduced tolerance toward uncertainties like internalizing disorders (e.g., anxiety disorder, major depressive disorder; Carleton, 2012; Mahoney and McEvoy, 2012), anorexia nervosa (Frank et al., 2012) and even ADHD (Gramszlo et al., 2018). Techniques based on rewards are an important component of behavioral therapy, and a better understanding of the reward system might have important implications for therapeutic procedures.

### Limitations and Future Directions

The present study includes limitations that warrant mention. The study used a cross-sectional design, thus not allowing conclusions to be drawn about development over time. Future studies would benefit from incorporating a longitudinal design with a larger sample size following participants from childhood to late adolescence. Second, it is possible that the colors of the cue stimuli could influence our results as we did not counterbalance the colors of the cue stimuli. As studies have demonstrated the context-dependent influence of colors on human behavior (Elliot and Maier, 2014), future studies should incorporate counterbalancing to limit such possible effects. Lastly, in contrast to the original MID task (Knutson et al., 2000), we did not include loss trials which might restrict our findings as reward trials may be anticipated differently when framed by loss trials (Reyna and Brainerd, 2011) compared to verbal trials. We decided to use this procedure for two reasons: First, we needed to ensure enough statistical power to compare the different conditions. Second, we had to keep the scanning period within a time frame that could be accomplished by adolescents to avoid excessive movement. Taken together, future studies could examine the effects of different reward probabilities by comparing a loss and verbal condition or by integrating all three conditions (reward, loss, and verbal) in one paradigm with a larger number of trials. As we observed a stronger neural reaction toward the verbal condition in adolescents compared to adults, future studies might further investigate whether the type of reward (e.g., money vs. more immediate forms of reward like gifts) modifies the neural response in the reward condition.

### Conclusion

This study offers a first insight into the processing of different reward probabilities in adults and adolescents. The current results demonstrated behavioral and neuronal differences between adolescents and adults such that both age groups reacted differently to varying reward probabilities. Whereas, adolescents showed faster reaction times and a higher percent signal change in the bilateral VS for the low reward probability (33%), adults demonstrate a higher percent signal change in the VS for the higher reward probabilities (66 and 88%). Further, in the higher reward probabilities the differences between the monetary and the verbal condition increased with age. This has implications for future research comparing adolescent and adult reward processing. Inconsistent findings in the literature comparing both age groups might result not only from different tasks but also from the reward probability that was used in the task. Since the present study focuses on the Monetary Incentive Delay Task which is only one of many paradigms used to examine the processing of rewards, it is an open question to what extent the present results can be generalized. It is therefore necessary to incorporate different paradigms in future studies. With that, it opens up the possibility to gain further insights into the transition from adolescence to young adulthood.

## Data Availability Statement

The raw data supporting the conclusions of this article will be made available by the authors, without undue reservation.

## Ethics Statement

The studies involving human participants were reviewed and approved by Ethics Committee, Faculty of Medicine, TU Dresden, Germany. Written informed consent to participate in this study was provided by the participants' legal guardian/next of kin.

## Author Contributions

JB, MP, and VR developed the study concept and design. MP programed the paradigm. Data collection was performed by MB. HW and MB developed the analysis pipeline. MB, NV, and JB performed the data analysis and interpreted the results. MB drafted the manuscript with substantial contribution of JB, and all other authors provided critical revisions and approved the final version of the manuscript for submission.

## Conflict of Interest

The authors declare that the research was conducted in the absence of any commercial or financial relationships that could be construed as a potential conflict of interest.
