# Supplementary material for: Ventral Striatal Activation During Reward Anticipation of Different Reward Probabilities in Adolescents and Adults
Source: Front Hum Neurosci. 2021 Apr 20;15:649724. doi: 10.3389/fnhum.2021.649724 (PMC8093817; doi:10.3389/fnhum.2021.649724)
Supplement: Supplementary file 1 [file Table_1.DOCX]

Supplementary Material

# Supplementary Data

S 1

| Error rates for both age groups (n = 47) | | | | | | | | | |
| --- | --- | --- | --- | --- | --- | --- | --- | --- | --- |
|  | Adolescents (n = 25) |  | Adults (n = 22) |  | Post-Hoc Tests - Comparison between groups | | | | |
|  | *M (SD) in ms* |  | *M (SD) in ms* |  | *Mean Difference* | *t* | *df* | *p_Scheffe_* | *d* |
| Total | 2.13 (*1.50*) |  | 1.22 (*0.74*) |  | -0.914 | -2.59 | 45 | .013 | -.756 |
| Monetary condition | 2.39 (*1.71*) |  | 1.57 (*1.14*) |  | -0.826 | -1.92 | 45 | .061 | -.562 |
| Verbal condition | 1.89 (*1.47*) *t*(24) = 2.41; *p* = .024 |  | 0.88 (*0.72*) *t*(21) = 2.67; *p* = .014 |  | -1.001 | -2.91 | 45 | .006 | -.851 |
| Reward Probability33 % |  |  |  |  |  |  |  |  |  |
| Total | 2.22 (*1.97*) |  | 1.36 (*0.88*) |  | -0.856 | -1.89 | 45 | .066 | -.551 |
| Monetary condition | 2.80 (*2.24*) |  | 1.77 (1.51) |  | -1.027 | -1.819 | 45 | .075 | -.532 |
| Verbal condition | 1.64 (1.93)  *t*(24) = - 4.04; *p* < .001 |  | 0.95 (1.00) t(21) = -2.05; p = .053 |  | -0.685 | -1.495 | 45 | .142 | -.437 |
| Reward Probability 66 % |  |  |  |  |  |  |  |  |  |
| Total | 2.20 (*1.69*) |  | 1.02 (*0.82*) |  | -1.177 | -2.970 | 45 | .005 | -.868 |
| Monetary condition | 2.20 (*1.94*) |  | 1.05 (*1.05*) |  | -1.155 | -2.493 | 45 | .016 | -.729 |
| Verbal condition | 2.20 (*1.89*) *t*(24) = .000; *p* = 1.00 |  | 1.00 (1.07) *t*(21) = -.161; *p* = .874 |  | -1.200 | -2.626 | 45 | .012 | -.768 |
| Reward Probability 88 % |  |  |  |  |  |  |  |  |  |
| Total | 1.98 (*1.60*) |  | 1.28 (*1.04*) |  | -0.707 | -1.77 | 45 | .084 | -.517 |
| Monetary condition | 2.16 (*2.21*) |  | 1.86 (*1.81*) |  | -0.296 | -.499 | 45 | .620 | -.146 |
| Verbal condition | 1.80 (*1.73*) *t*(24) = -.762; *p* = .453 |  | 0.68 (*0.94*) *t*(21) = -2.78; *p* = .011 |  | -1.118 | -2.693 | 45 | .010 | -.787 |
